# Principles of Forensic Medicine

**Published:** 1846-03-01

**Authors:** 


*Principles of Forensic Medicine, by Wm. A. Guy, M. B., Cantab, etc.—First American edition, with notes and additions, by Charles A. Lee, M. D., etc.*, 1 *vol.* 8 *mo.* 700 *pp.*

The advantage of being familiar with the various subjects embraced in Forensic Medicines, or medical jurisprudence, is frequently not fully appreciated until the practitioner is unexpectedly called upon to investigate or testify in a medico-legal case involving great responsibility. Then, if this department of medical science have been neglected, the practitioner is often made to experience proofs of its necessity which are as mortifying as convincing. We are all liable at any moment to be placed in a position where we can only escape responsibility by pleading ignorance, or must pronounce opinions upon which depend property, character and even life. In view of the considerations no medical man should be without one or more standard treatises on this branch. That of Prof. Beck is one of the most elaborate and complete, and it is a matter of just pride to the profession of this country that one of its members should have produced a work which is respected as of the highest authority in other countries, not less than in our own. Inasmuch as we desire to see the medical literature of our country developed and cherished, in so far as we could exert an influence it would be to give in all cases preference in our patronage to American publications, provided they are not inferior in merit. The work of Beck should be in the library of every Physician who can afford to purchase it. Other treatises, however, may advantageously be added, or in case of the necessity of being governed by the *res angusta*, those substituted which are afforded at a cheaper price. The work whose title page is prefixed, has lately been issued from the press of Harper & Brothers, New-York.—It presents a comprehensive summary of the important facts and principles involved in the various classes of medico-legal questions, omitting the discussion of points which possess interest in a literary point of view only, and devoting attention to what is of direct practical utility. So far as we can judge, it is admirably adapted to serve as a text-book, or manual for the practitioner, and as such, we cordially recommend it. The office of editorship in this instance has not been merely a nominal one. The extensive additions contributed by Dr. Lee constitute it, to a considerable extent, an American work. These are not less interesting and valuable than the context, and are adapted to meet all the wants of the American reader.

The typographical appearance is highly creditable to the publishers; and its price ($3,00) considering its size and execution is extremely reasonable.

